# Two single nucleotide polymorphisms sites in *α1*-*AT* gene and their association with somatic cell score in Chinese Holstein cows

**DOI:** 10.1186/s40709-017-0065-z

**Published:** 2017-04-13

**Authors:** Xiao-Fei Guo, Wen-Ping Hu, Xian-Zheng Lang, Qiu-Ling Li, Xiang-Yu Wang, Ran Di, Qiu-Yue Liu, Xiao-Lin Liu, Yong-Fu An, Ming-Xing Chu

**Affiliations:** 1 Key Laboratory of Animal Genetics and Breeding and Reproduction of Ministry of Agriculture, Institute of Animal Science, Chinese Academy of Agricultural Sciences, Beijing, 100193 China; 2 College of Animal Science and Technology, Northwest A &F University, Yangling, 712100 China; 3 Life Sciences College, Langfang Teachers University, Langfang, 065000 China; 4 Hebei Animal Science and Veterinary Medicine Institute, Baoding, 071000 China; 5 College of Animal Science and Technology, China Agricultural University, Beijing, 100193 China

**Keywords:** SNP, *α1*-*AT* gene, Polymorphism, Somatic cell score, Chinese Holstein cows

## Abstract

**Background:**

Alpha 1-antitrypsin (α1-AT) may affect the susceptibility of mastitis in dairy cattle for its possible role in the protection of lactoferrin from proteolytic degradation in the mammary. Milk somatic cell score (SCS) is a logarithmic transformation of the milk somatic cell count widely used as an index to evaluate mastitis. To study the relationships of *α1*-*AT* gene and SCS in Chinese Holstein cows, methods of PCR-SSCP, DNA sequencing, PCR-RFLP, and CRS-PCR technologies were used to detect single nucleotide polymorphisms sites in *α1*-*AT* gene.

**Results:**

Two polymorphic sites at G5503A and G5746C of *α1*-*AT* gene were found. AA (0.3633), AB (0.4644) and BB (0.1723) genotypes were detected at G5503A site, CC (0.3483), CD (0.4906) and DD (0.1611) genotypes were found at G5746C in Chinese Holstein cows. Least squares mean of SCS for individuals with BB genotype was significantly lower than that with AA and AB genotype (*p* < 0.01), and that with AB genotype was significantly lower than that with AA (*p* < 0.05). There was no significant difference among individuals with CC, CD and DD genotypes (*p* > 0.05). Least squares mean of SCS for individuals with BBDD genotype combination were significantly lower than those with AACC and AACD (*p* < 0.05).

**Conclusions:**

Statistical analysis indicated that B allele and BBDD genotype combination of α1-AT can improve mastitis resistance in dairy cattle.

## Background

Alpha 1-antitrypsin (α1-AT) or alpha 1-protease inhibitor (α1-PI), is mainly synthesized in the animals’ liver while it exists in plants and microorganisms, as well. As a member of serine protease inhibitor protein subfamily, α1-AT inhibits the target proteases by a specific mechanism, which depends on a change in conformation [1]. The α1-AT could be isolated from cow’s milk, its molecular size ranges from 56 to 64 kDa and it possesses the characteristic of cell membrane permeability. The α1-AT can suppress the reaction of trypsin and elastase, while it is not active against plasmin [2]. Chowanadisai and Lönnerdal [3] had reported that α1-AT was expressed in mammary gland epithelial cells, and it can protect lactoferrin from digestion by pancreatin in vitro, which suggested that it had the potential of protecting milk proteins by inhibiting proteases in the gastrointestinal tract of infants. The α1-AT might be the predominant protease inhibitor in milk, for its ability to inhibit both trypsin and chymotrypsin, which are the primary pancreatic proteases [3]. The α1-AT protein in bovine milk may inhibit the hydrolysis of trypsin on lactoferrin [4, 5]. Heihavand-Kheiripour et al. [6] suggested that α1-AT protein may affect the susceptibility of mastitis in dairy cattle for its possible role in the protection of lactoferrin from proteolytic degradation in the mammary.

The bovine *α1*-*AT* gene is located on chromosome 21 (approximately 9 kb of genomic DNA), consists of five exons, and encodes a protein with 416 amino acids [7]. Sinha et al. [8] had isolated a whole cDNA clone coding for bovine α1-AT from a λgt11 bovine liver cDNA library using a human α1-AT cDNA as a probe. Five single nucleotide polymorphisms (SNPs) in coding regions of the bovine *α1*-*AT* gene were found by direct sequencing of reverse transcription-polymerase chain reaction (RT-PCR) products from a wide range of cattle tissues. The relationship of these SNPs with the economic traits in North American Holstein population was studied [9]. Meanwhile, researchers had reported that *α1*-*AT* gene was associated with milk production traits in dairy cattle [10–12].

Since it is difficult to measure the mastitis phenotype using a direct index, milk SCS has been most widely used as an index to evaluate mastitis [13]. Milk SCS is a logarithmic transformation of the milk somatic cell count (SCC) to achieve normality of distribution in statistics which has positive correlation with clinical mastitis [14–17]. In the present study, two single nucleotide polymorphisms sites of *α1*-*AT* gene were found in Chinese Holstein cows and their relationships with SCS were also studied; the results may provide a theoretical basis for marker-assisted selection of mastitis resistance in Chinese Holstein cows.

## Results

### PCR amplification and SSCP detection

The *α1*-*AT* gene in 267 Chinese Holstein cows was amplified using the designed primers (Table 1). The results showed that the amplification fragment sizes were consistent with the target ones. The PCR products of three pairs of primers were all analyzed by SSCP. Polymorphisms were only found in the amplified fragments in the results of P1 and P3 primers. P1 and P3 primers were adopted for following analysis.

**Table 1:** Primer sequence, product size, location and annealing temperature of *α1*-*AT* gene in Chinese Holstein cows

Primer	Primer sequence (5′ → 3′)	Product size (bp)	Location^a^	Annealing temperature (°C)
P1	F: GCCATTGTTCTGAGTCTTTC	978	5216–5235	54
	R: TTCCCTAACCCTATTTGATT		6174–6193	
P2	F: GGCACCAACTGGAAAGAACAAC	175	8060–8081	59
	R: AGCCCTATCGCTGAAGACCT		8215–8234	
P3	F: CCTTTGCGATGCTCTCCCTG	149	5616–5635	55
	R: CTGGTGGTTTGGCTGATT		5747–5764	

^a^Base positions corresponding to NC_007319 of GenBank

## Sequencing analysis

**Fig. 1:**
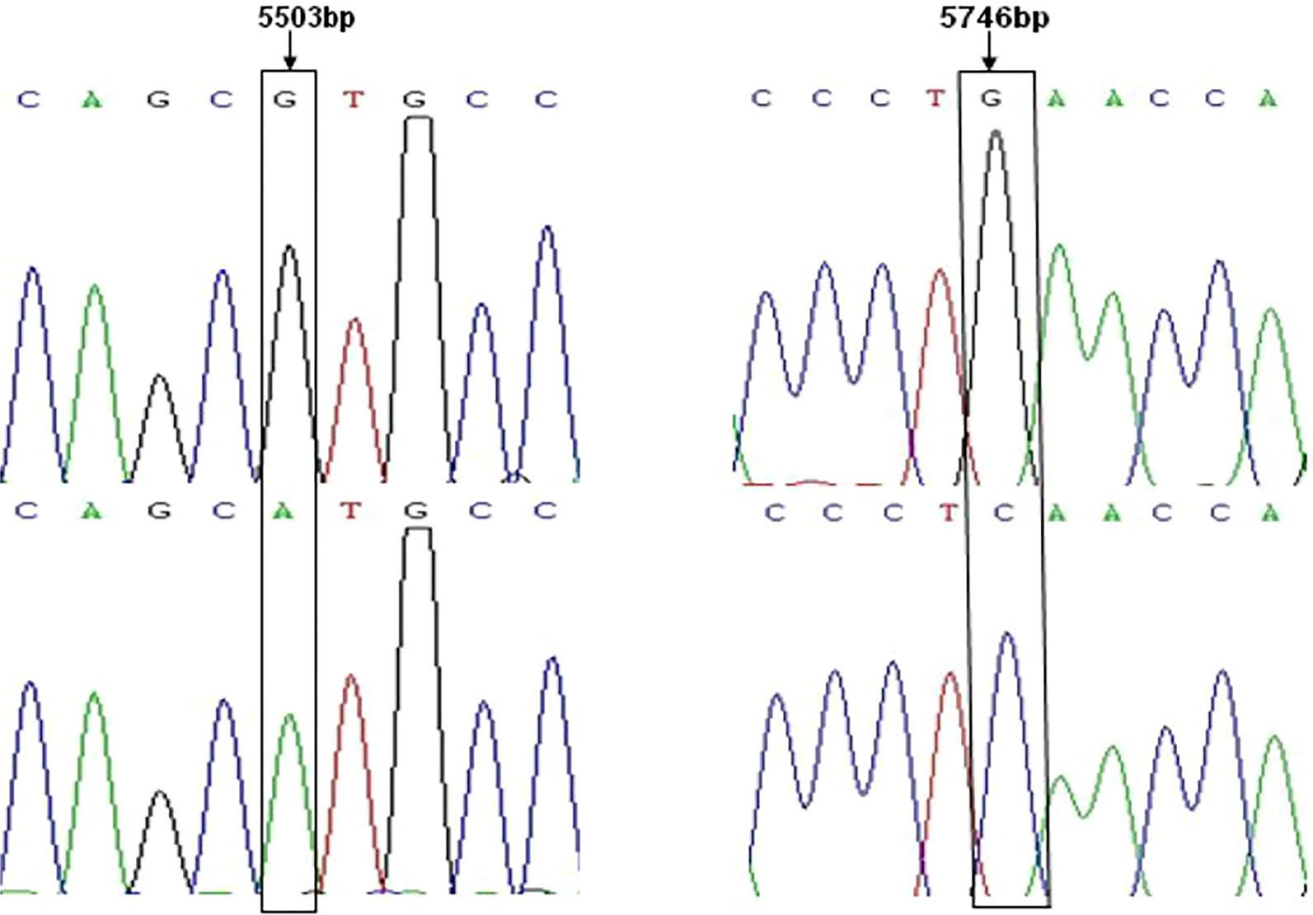
Two SNPs of *al*-*AT* gene in Chinese Holstein cows

The products of primer P1, P3 were cloned and sequenced. The sequences were compared by DNAMAN software (Lynnon Biosoft Inc., San Ramon, USA). In the second exon of *α1*-*AT* gene, one mutation of G → A was found at the 5503 bp site, which generated the *Sph*I restriction enzyme site and caused no amino acid change. Another mutation of G → C was found at the 5746 bp site, which also caused no amino acid change (Fig. 1).

### RFLP detection

#### *Sph*I analysis of the G5503A site

**Fig. 2:**
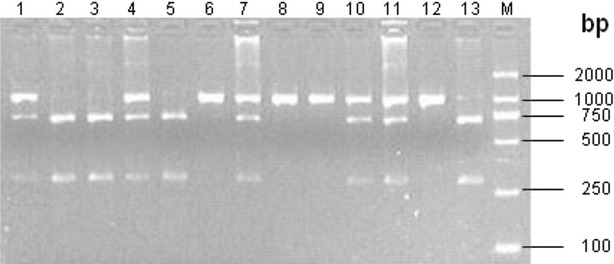
*Sph*I-RFLP patterns of PCR products of G5503A site in 2% agarose gel. *Note* 6, 8, 9, 12: AA genotype; 1, 4, 7, 10, 11: AB genotype; 2, 3, 5, 13; BB genotype; M: D2000 DNA marker

The 978 bp PCR products were completely digested with *Sph*I restriction endonuclease and genetic polymorphisms of *α1*-*AT* were investigated by PCR-RFLP. As shown in Fig. 2, three genotypes, AA (978 bp), AB (978/688/290 bp) and BB (688/290 bp), were found in 267 Chinese Holstein cows.

#### *Hinf*I analysis of the G5746C site

**Fig. 3:**
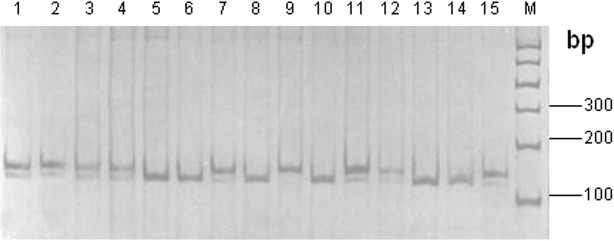
*Hinf*I-RFLP patterns of PCR products of G5746C site in 12% polyacrylamide gel. *Note* 5, 6, 8, 10, 13, 14: CC genotype; 1–4, 7, 11, 12, 15: CD genotype; 9: DD genotype; M: DNA marker I

Because of no restriction enzyme site existing in G5746C, the restriction enzyme site of *Hinf*I was introduced by created restriction site-PCR (CRS-PCR) based on primer P3. The 149 bp PCR products were digested by *Hinf*I restriction endonuclease; then, the genetic polymorphisms could be detected by PCR-RFLP. As shown in Fig. 3, three genotypes, CC (131 bp), CD (149/131 bp) and DD (149 bp), were found in 267 Chinese Holstein cows.

### Frequencies of allele and its genotype

The frequencies of alleles and genotypes of *α1*-*AT* gene in experimental Holstein cows were presented in Table 2. At locus of G5503A and G5746C, the frequencies of AA, AB, BB and CC, CD, DD genotypes were 0.3633, 0.4644, 0.1723 and 0.3483, 0.4906, 0.1611, respectively, while the frequencies of A, B, C and D allele were 0.5955, 0.4045, 0.5936 and 0.4064, respectively. Chi square fitness test of the mutation site showed that the locus of G5503A and G5746C were in Hardy–Weinberg equilibrium in these Chinese Holstein cows (*p* > 0.05). Polymorphism Information Content (PIC) of the polymorphic sites G5503C and G5746C were >0.3, indicative of moderate polymorphism; this finding implies that the selection potential of the sites was relatively large (Table 3).

**Table 2:** Allele and genotype frequencies of *α1*-*AT* gene in Chinese Holstein cows

Polymorphic site	Genotype	Number of samples	Genotype frequency	Allele	Allele frequency
G5503A	AA	97	0.3633	A	0.5955
	AB	124	0.4644	B	0.4045
	BB	46	0.1723		
G5746C	CC	93	0.3483	C	0.5936
	CD	131	0.4906	D	0.4064
	DD	43	0.1611

**Table 3:** Genetic characteristics of two polymorphic sites in Chinese Holstein cows

Polymorphic site	χ^2^ (*p*)	Polymorphism information content	Heterozygosity	Effective number of alleles
G5503A	0.35 (0.841)	0.366	0.482	1.93
G5746C	0.00 (0.999)	0.367	0.485	1.94

### Genotype combination analysis

The two polymorphic sites have generated nine genotype combinations (Table 4). The frequencies of AACC, AACD, AADD, ABCC, ABCD, ABDD, BBCC, BBCD and BBDD were 0.2697, 0.0899, 0.0075, 0.0749, 0.3408, 0.0412, 0.0037, 0.0599 and 0.1124, respectively. Further analysis showed that these nine genotype combinations were composed of the four haplotypes of A + C, A + D, B + C and B + D, the frequency of haplotype A + D was the lowest, while that of the A + C haplotype was the highest.

**Table 4:** Frequencies of genotype combinations of the two polymorphic sites

Genotype combination	Genotype combination frequencies
AACC	72 (0.2697)
AACD	24 (0.0899)
AADD	2 (0.0075)
ABCC	20 (0.0749)
ABCD	91 (0.3408)
ABDD	11 (0.0412)
BBCC	1 (0.0037)
BBCD	16 (0.0599)
BBDD	30 (0.1124)

### The relationship between the polymorphism of *α1*-*AT* gene and SCS in Chinese Holstein cows

For the polymorphic sites of G5503A and G5746C, the results of variance analysis indicated that the bull, herd, parity and calving season had a significant effect on SCS (*p* < 0.05). Least squares means and standard errors for SCS of different genotypes and genotype combinations were listed in Tables 5 and 6. The results showed that the least squares mean of SCS of BB genotype was significantly lower than that of AA and AB genotypes (*p* < 0.05), and the least squares mean of SCS of AB genotype was significantly lower than that of AA genotype (*p* < 0.05). The least squares means of SCS had no significant difference between CC, CD and DD genotypes (*p* > 0.05). The result of variance analysis of genotype combinations showed that the least squares mean of SCS of BBDD genotype combination was significantly lower than that of AACC and AACD genotype combinations (*p* < 0.05). In regard to mastitis resistance, BB was the most favorable genotype, AA was the most unfavorable genotype, BBDD was the most favorable genotype combination, and AACC was the most unfavorable genotype combination. The results preliminarily indicated that allele B of G5503A polymorphic site of *α1*-*AT* gene is a potential DNA marker for improving mastitis resistance in Chinese Holstein cow.

**Table 5:** Least square means (LSM) and standard error (SE) for somatic cell scores of different genotypes

Polymorphic site	Genotype	Number of samples	Somatic cell score
G5503A	AA	97	4.27 ± 0.13^a^
	AB	124	3.81 ± 0.10^b^
	BB	46	3.29 ± 0.18^c^
G5746C	CC	93	3.97 ± 0.15^a^
	CD	131	3.91 ± 0.11^a^
	DD	43	3.65 ± 0.20^a^

Least squares means with the same superscript for the same site have no significant difference (*p* > 0.05). Least squares means with the different superscripts for the same site differ significantly (*p* < 0.05)

**Table 6:** Least squares mean (LSM) and standard error (SE) for somatic cell scores of different genotype combinations

Genotype combination	Number of samples	Somatic cell score
AACC	72	4.15 ± 0.16^a^
AACD	24	4.05 ± 0.22^a^
AADD	2	
ABCC	20	3.89 ± 0.23^ab^
ABCD	91	3.84 ± 0.15^ab^
ABDD	11	3.75 ± 0.24^ab^
BBCC	1	
BBCD	16	3.71 ± 0.24^ab^
BBDD	30	3.43 ± 0.21^b^

Least squares means with the same superscript for the same site have no significant difference (*p* > 0.05). Least squares means with the different superscripts for the same site differ significantly (*p* < 0.05)

## Discussion

Like the other traits in cows, yield and quality of milk were affected by factors of heredity, nutrition and environment [18–21]. At genetic level, several molecular marker genes which associated with milk production traits were detected in various dairy cows over the years [22–26]. As one of the marker genes, five SNPs of the *α1*-*AT* gene (at positions 164, 269, 284, 407 and 989 bp on mRNA) were identified in North American Holsteins by Khatib et al. [9]. Researchers found that haplotype D was associated with a significant increase in milk, fat percentage and protein percentage, while haplotypes B and C were associated with an increase in milk protein percentage [9, 27]. The 668 and 999 bp fragments of 5′ flanking region in the *α1*-*AT* gene were amplified in Chinese Holsteins, and the C-T and T-C SNPs were identified at nucleotide +3142 and +4408 bp by sequencing, in which, the allele B had a significant impact on milk yield and fat/protein ratio, and the allele F had a significant influence on milk fat percentage in Chinese Holstein cows [28].

In the present study, two mutations of G5503A and G5746C in *α1*-*AT* gene were detected in Chinese Holstein cows, which were consistent with the mutations at 164 and 407 bp in its mRNA, while the mutations at 269, 284 and 989 bp were not detected. The two mutations in this study caused no amino acid change, similar to studies made by other authors [6, 10]. It should be noted that, although SNPs in *α1*-*AT* gene were not functional mutations, they were possibly in linkage disequilibrium with a certain functional polymorphic site in the *α1*-*AT* gene or other closely linked gene(s) influencing milk-related traits [6, 7].

Over the years, most dairy breeding goals aim at increasing the milk production by high-strength artificial selection while ignored health traits such as mastitis resistance. A higher yielding cow results in a higher risk of mastitis, which would lead to an involuntary and premature culling of milking cows for defective udder characteristics and decreased milk yield [13, 14]. In the breeding of cattle, dairy breeds were less resistant to mastitis than dual-purpose breeds [29, 30]. The *α1*-*AT* gene was treated as a candidate gene of milk production trait, since α1-AT can protect breast tissue and milk protein from excessive hydrolysis damage. In addition, as a kind of multifunctional serine proteinase inhibitor, α1-AT protein also displays a wide range of anti-inflammatory properties [31]. Cow mastitis could cause the increased permeability between the blood and breast milk. Large amounts of neutrophil elastases could be secreted to decompose the pathogenic bacteria. As a marker protein of a cow with mastitis, the concentration of α1-AT protein will be higher than the health individuals. For the protective mechanism for the body of α1-AT protein, it was supposed that the somatic cell count in milk might be associated with the content of α1-AT protein in serum.

## Conclusions

The relationship between the two single nucleotide polymorphisms sites of *α1*-*AT* gene and SCC/SCS in Chinese Holstein cow milk was explored in the present study. The relationships between the mutations of G5503A and G5746C with SCS in Chinese Holstein cow were analyzed by least square method, and the results showed that the genotype combination of BBDD maybe a favorable combination for mastitis resistance, which means that B allele and BD haplotype in this study can be used as the genetic markers for mastitis resistance in Chinese Holstein cows.

Further studies focused on associating the various polymorphisms in *α1*-*AT* gene with SCS in different regions and populations of Holstein cows are necessary to be carried out. Based on these results, further progress could be made to determine whether the *α1*-*AT* gene can be recognized as the candidate gene of mastitis resistance.

## Methods

### Blood sample collection and DNA preparation

In 2015, jugular blood samples (10 ml per cow) were collected from a total of 267 Chinese Holstein cows calved (the first parity, n = 85; the second parity, n = 90; the third parity, n = 92), originated from four dairy farms (79 cows from 1st farm, 71 cows from 2nd farm, 61 cows from 3rd farm and 56 cows from 4th farm, respectively) in Hebei province, P. R. China, using acid citrate dextrose as an anticoagulant. Genomic DNA was extracted from whole blood by the phenol–chloroform method, and then dissolved in TE buffer [10 mmol l^−1^ Tris–HCl (pH 8.0), 1 mmol l^−1^ EDTA (pH 8.0)] and kept at −20 °C.

The 267 Chinese Holstein cows were selected from the progeny of five bulls at random, and each number of progeny of the five bulls was 50, 51, 52, 55 and 59, respectively. Calving was partitioned into four 3-month seasons: March through to May (season 1, spring, n = 70), June through to August (season 2, summer, n = 63), September through to November (season 3, autumn, n = 76) and December through to February (season 4, winter, n = 58).

SCC in milk samples were estimated by MilkoScan (FOSS 6000, Denmark) with unit of cells μl^−1^. SCS was measured according to the calculation formula: $SCS = \left( {\log_{2} \frac{SCC}{100000}} \right) + 3$.

### Primer design

Three pairs of primers were designed according to bovine *α1*-*AT* gene sequence (GenBank accession number NC_007319) using Primer 5.0 and Oligo 6.0 software, and synthesized by Shanghai Invitrogen Biotechnology Co., Ltd.

### Cloning and sequencing

Fifteen different individuals were selected randomly for PCR amplification. The PCR products were rapidly recovered by DNA purification kit, and the DNA fragments were inserted into pGEM-T Easy vector (Promega, Madison, WI, USA) according to the manufacturer’s instructions. The recombinant plasmid was transformed into competent *Escherichia coli* DH5α; then, the positive clones were identified and sent to Beijing Jin Wei Zhi Company for sequencing.

### PCR-SSCP detection

Polymerase chain reaction (PCR) was carried out in 25 μl volume containing 2.5 μl × 10 PCR buffer, 1.0 μl of each primer (forward and reverse, 10 μM), 2.5 μl MgCl_2_ (20 mM), 2.0 μl dNTPs (2 mM), 1.0 μl *Taq* DNA polymerase (2.5 U μl^−1^, SABC, Beijing, China), 3.0 μl DNA template (50 ng μl^−1^) and 12 μl of dH_2_O. Amplification conditions were as follows: initial denaturation at 94 °C for 5 min followed by 35 cycles of denaturation at 94 °C for 60 s, annealing at 59 °C for 60 s, extension at 72 °C for 15 s with a final extension step at 72 °C for 10 min on Mastercycler^®^ 5333 (Eppendorf AG, Hamburg, Germany).

PCR products of primer P2 were used for SSCP analysis. A volume of 3 μl PCR product was transferred into the Eppendorf tube, mixed with 7 μl gel loading solution containing 98% formamide, 0.025% bromophenol blue, 0.025% xylene cyanol, 20 mmol l^−1^ EDTA (pH 8.0) and 10% glycerol. The mixture was centrifuged and denaturated at 98 °C for 10 min, then chilled on ice for 7 min and loaded on 10-12% neutral polyacrylamide gels (acrylamide:bisacrylamide = 29–39:1). Electrophoresis was performed in × 1 Tris borate (pH 8.3)-EDTA buffer at 9–15 V cm^−1^ for 14–16 h at 4 °C. After electrophoresis, the DNA fragments in the gels were visualized by silver staining, photographed and analyzed using an AlphaImager™ 2200 and 1220 Documentation and Analysis Systems (Alpha Innotech Corporation, San Leandro, CA, USA).

### Restriction fragment length polymorphism (RFLP) analysis

Restriction enzyme reaction system mixture (10 μl volume) were incubated at 37 °C overnight (contained 3 μl of PCR products, 0.5 μl restriction enzyme, 1 μl corresponding ×10 reaction buffer, 5.5 μl ultrapure H_2_O). The enzyme-digested products were visualized by 2% agarose gel electrophoresis, and identified by silver nitrate staining, then photographed and analyzed.

Based on the results of sequence of P1, one mutation was revealed in exon 3 and restriction enzyme *Sph*I was used to detect it. The PCR products of P3 were digested by *Sph*I (NEB, Beijing, China) with 10 μl volume containing 5 μl of PCR product, 0.5 μl of 10 U µl^−1^ restriction enzyme *Sph*I, 1 μl of ×10 reaction buffer, 3.5 μl H_2_O, incubated at 37 °C for 5 h. The mixtures were detected by 3.0% agarose gels and were genotyped.

### Creating restriction site (CRS)

When there was no available restriction enzyme site for PCR-RFLP analysis, CRS combined with PCR amplification is a simple and efficient method that could be used to detect single nucleotide polymorphisms (SNPs) genotypes [8]. One-mismatch bases are used in a primer P3 in this study to create a restriction site for PCR. Then the PCR products can be genotyped in the same way as RFLP.

### Statistical analysis

A mutation was assumed that appearing on a base site, and the computational formula of genotype frequency in resultant three genotypes (AA, AB, BB) were as follows:

$$ f(AA) = \frac{{n_{AA} }}{{n_{AA} + n_{AB} + n_{BB} }};\quad f(AB) = \frac{{n_{AB} }}{{n_{AA} + n_{AB} + n_{BB} }};\quad f(BB) = \frac{{n_{BB} }}{{n_{AA} + n_{AB} + n_{BB} }} $$

note: n_AA_ is the number of AA, n_AB_ is the number of AB, n_BB_ is the number of BB.

Computational formula of allele frequency:

$$ p = \frac{{2n_{AA} + n_{AB} }}{{2(n_{AA} + n_{AB} + n_{BB} )}};\quad q = \frac{{2n_{BB} + n_{AB} }}{{2(n_{AA} + n_{AB} + n_{BB} )}} $$

note: *p* is the allele frequency of A, *q* is the allele frequency of B.

Computational formula of heterozygosity (*He*):

$$ He = 1 - \mathop \sum \limits_{i = 1}^{n} p_{i}^{2} $$

note: n is the number of allele, *p*_*i*_ is the allele frequency of the *i*th allele.

Computational formula of effective number of alleles (*Ne*):

$$ Ne = 1/\mathop \sum \limits_{i = 1}^{n} p_{i}^{2} $$

note: n is the number of allele, *p*_*i*_ is the allele frequency of the *i*th allele.

Computational formula of Polymorphism Information Content (*PIC*):

$$ PIC = 1 - \mathop \sum \limits_{i = 1}^{n} p_{i}^{2} - \mathop \sum \limits_{i = 1}^{n - 1} \mathop \sum \limits_{j = i + 1}^{n} 2p_{i}^{2} p_{j}^{2} $$

note: n is the number of allele, *p*_*i*_ is the allele frequency of the *i*th allele, and *p*_*j*_ is the allele frequency of the *j*th allele.

The following statistical model was fitted to compare difference of SCS among different genotypes in Chinese Holstein cows.

$$ y_{ijklmn} = \, \mu \, + \, B_{i} + \, H_{j} + \, P_{k} + \, CS_{l} + \, G_{m} + \, e_{ijklmn} $$

where *y*_*ijklmn*_ is phenotypic value of SCS; *μ* is population mean; *B*_*i*_ is the fixed effect of the *i*th bull (*i* = 1, 2, 3, 4, 5); *H*_*j*_ is the fixed effect of the *j*th herd (*j* = 1, 2, 3, 4); *P*_*k*_ is the fixed effect of the *k*th parity (*k* = 1, 2, 3); *CS*_*l*_ is the fixed effect of the *l*th calving season (*l* = 1, 2, 3, 4); *G*_*m*_ is the fixed effect of the *m*th genotype (*m* = 1, 2, 3); *e*_*ijklmn*_ is the random error effect of each observation. Analysis was performed using the general linear model procedure of SAS (Ver 8.12) (SAS Institute Inc., North Carolina, USA). Mean separation procedures were performed using a least significant difference test.

## Abbreviations

α1-AT: alpha 1-antitrypsin
SCS: somatic cell score
SNPs: single nucleotide polymorphisms
PCR: polymerase chain reaction
RT-PCR: reverse transcription-polymerase chain reaction
SCC: somatic cell count
RFLP: restriction fragment length polymorphism
CRS: creating restriction site
PIC: polymorphism information content

## References

[1] Bots M, Medema JP (2008). Serpins in T cell immunity. J Leukoc Biol.

[2] Weber BA, Nielsen SS (1991). Isolation and partial characterization of a native serine-type protease inhibitor from bovine milk. J Dairy Sci.

[3] Chowanadisai W, Lönnerdal B (2002). α1-antitrypsin and antichymotrypsin in human milk: origin, concentrations, and stability. Am J Clin Nutr.

[4] He XW, Liu Y, Luo ZG (2006). Alpha1 antitrypsin and its clinic application. Acad Period Farm Prod Process..

[5] Zhuang MQ, Wu MP, Ai ZW, Qin L, Zhang J, Sheng FX (2008). Isolation and purification of α1-antitrypsin. J Microbiol..

[6] Heihavand-Kheiripour M, Mahdavi AH, Rahmani HR, Soltani-Ghombavani M, Edriss MA (2014). Association of polymorphism in the alpha-1-antitrypsin gene with milk production traits in Holstein dairy cows. S Afr J Anim Sci..

[7] Khatib H (2005). Monoallelic expression of the protease inhibitor gene in humans, sheep, and cattle. Mamm Genome.

[8] Sinha D, Bakhshi MR, Kirby EP (1992). Complete cDNA sequence of bovine alpha 1-antitrypsin. Biochim Biophys Acta.

[9] Khatib H, Heifetz E, Dekkers JC (2005). Association of the protease inhibitor gene with production traits in Holstein dairy cattle. J Dairy Sci.

[10] Li QL, Zhang ZF, Wang CF, Yang H, Wang HM, Li JB (2010). Association of polymorphism of the alpha 1-antitrypsin gene with milk production traits in Chinese Holstein. S Afr J Anim Sci..

[11] Mosig MO, Lipkin E, Khutoreskaya G, Tchourzyna E, Soller M, Friedmann A (2001). A whole genome scan for quantitative trait loci affecting milk protein percentage in Israeli-Holstein cattle, by means of selective milk DNA pooling in a daughter design, using an adjusted false discovery rate criterion. Genetics.

[12] Rodriguez-Zas SL, Southey BR, Heyen DW, Lewin HA (2002). Interval and composite interval mapping of somatic cell score, yield, and components of milk in dairy cattle. J Dairy Sci.

[13] Yuan ZR, Li J, Liu L, Zhang LP, Zhang LM, Chen C (2011). Single nucleotide polymorphism of CACNA2D1 gene and its association with milk somatic cell score in cattle. Mol Biol Rep.

[14] Alam M, Cho CI, Choi TJ, Park B, Choi JG, Choy YH (2015). Estimation of genetic parameters for Somatic Cell Scores of Holsteins using multi-trait lactation models in Korea. Asian-Australas J Anim Sci..

[15] Ali AKA, Shook GE (1980). An optimum transformation for somatic cell concentration in milk. J Dairy Sci.

[16] Rupp R, Boichard D (1999). Genetic parameters for clinical mastitis, somatic cell score, production, udder type traits, and milking ease in first lactation Holsteins. J Dairy Sci.

[17] Holmbeg M, Andersson-Eklund L (2004). Quantitative trait loci affecting health traits in Swedish dairy cattle. J Dairy Sci.

[18] Abdalla EA, Weigel KA, Byrem TM, Rosa GJ (2016). Short communication: genetic correlation of bovine leukosis incidence with somatic cell score and milk yield in a US Holstein population. J Dairy Sci.

[19] Subhadra B (2011). Grinson-G. Algal biorefinery-based industry: an approach to address fuel and food insecurity for a carbon-smart world. J Sci Food Agric.

[20] Yaakob Z, Ali E, Zainal A, Mohamad M, Takriff MS (2014). An overview: biomolecules from microalgae for animal feed and aquaculture. J Biol Res (Thessalon)..

[21] Mote SS, Chauhan DS, Ghosh N (2016). Effect of environment factors on milk production and lactation length under different seasons in crossbred cattle. Indian J Anim Res..

[22] Szewczuk M (2017). Polymorphism in exon 2 encoding the putative ligand binding pocket of the bovine insulin-like growth factor 1 receptor affects milk traits in four different cattle breeds. J Anim Breed Genet.

[23] Vanbergue E, Peyraud JL, Guinard-Flament J, Charton C, Barbey S, Lefebvre R (2016). Effects of DGAT1 K232A polymorphism and milking frequency on milk composition and spontaneous lipolysis in dairy cows. J Dairy Sci.

[24] Bovenhuis H, Visker MH, Poulsen NA, Sehested J, van Valenberg H, van Arendonk JA (2016). Effects of the diacylglycerol o-acyltransferase 1 (DGAT1) K232A polymorphism on fatty acid, protein, and mineral composition of dairy cattle milk. J Dairy Sci.

[25] Wang X, Xu SZ, Gao X, Li JY, Ren HY, Luoren ZM (2008). Cloning and SNP screening of the TLR4 gene and the association between its polymorphism and somatic cell score in dairy cattle. S Afr J Anim Sci..

[26] Ramesha KP, Rao A, Basavaraju M, Geetha GR, Kataktalware MA, Jeyakumar S (2015). Genetic variability of bovine GHR, IGF-1 and IGFBP-3 genes in Indian cattle and buffalo. S Afr J Anim Sci..

[27] Heusipp G, Spekker K, Brast S, Fälker S, Schmidt MA (2006). YopM of *Yersinia enterocolitica* specifically interacts with α1-antitrypsin without affecting the anti-protease activity. Microbiology.

[28] Bu DQ, Li QL, Wang CF, Wang HM, Huang JM, Li JB (2009). Identification of new SNPs in bovine α1-antitrypsin gene 5′ flanking region and their association with milk production traits in Chinese Holstein dairy cattle. Chin J Biochem Mol Biol..

[29] Zhang LP, Gan QF, Ma TH, Li HD, Wang XP, Li JY (2009). Toll-like receptor 2 gene polymorphism and its relationship with SCS in dairy cattle. Anim Biotechnol..

[30] Wang XP, Xu SZ, Gao X, Ren HY, Chen JB (2007). Genetic polymorphism of TLR4 gene and correlation with mastitis in cattle. J Genet Genomics..

[31] Song S, Goudy K, Campbell-Thompson M, Wasserfall C, Scott-Jorgensen M, Wang J (2004). Recombinant adeno-associated virus-mediated alpha-1 antitrypsin gene therapy prevents type I diabetes in NOD mice. Gene Ther.

